# Efecto del síndrome de cabeza adelantada en el desarrollo de trastornos temporomandibulares

**DOI:** 10.21142/2523-2754-1004-2022-133

**Published:** 2022-12-26

**Authors:** Jordi Tomás, Carolina Castillo, Gabriela Villarroel, Álvaro Giner, Natalia Felipe

**Affiliations:** 1 Programa de maestría en Disfunción Craneomandibular y Dolor Orofacial, Universitat Internacional de Catalunya. Barcelona, España. jtomas@uic.es, ccastillobom@gmail.com, gabyvilla@uic.es, a_giner91@hotmail.com, nfelip@uic.es Universitat Internacional de Catalunya Programa de maestría en Disfunción Craneomandibular y Dolor Orofacial Universitat Internacional de Catalunya Barcelona Spain jtomas@uic.es ccastillobom@gmail.com gabyvilla@uic.es a_giner91@hotmail.com nfelip@uic.es

**Keywords:** temporomandibular, postura, cabeza, mialgia, puntos gatillo, temporomandibular, posture, head, myalgia, trigger points

## Abstract

Los malos hábitos, como la mala postura durante el uso de dispositivos tecnológicos, el mal control abdominal y el patrón de respiración oral, pueden conducir a una posición de la cabeza adelantada, lo que tiene implicaciones importantes en todo el devenir del individuo, especialmente a nivel de sistema estomatognático. Existe una fuerte asociación entre los trastornos temporomandibulares y el síndrome de cabeza adelantada (SCA). En estos casos, el componente muscular es el principal afectado, con la aparición de más puntos gatillo a nivel de los músculos esternocleidomastoideo, trapecio superior, *rectus capitis posterior* y *obliquus capitis superior*. Se han observado cambios degenerativos en la articulación temporomandibular (ATM), pero aún falta evidencia para asegurar que el síndrome de cabeza adelantada sea el factor etiológico.

## Abstract (en)

Bad habits, such as poor posture during the use of technological devices, poor abdominal control and oral breathing pattern can lead to a forward head position, which has important implications for the entire future of the individual, especially at the stomatognathic system. There is a strong association between temporomandibular disorders and forward head syndrome. Where the muscular component is the main affected, with the appearance of more trigger points at the level of the sternocleidomastoid muscles, upper trapezius, rectus capitis posterior and upper oblique capitis. Degenerative changes in the temporomandibular joint have been observed, but evidence is still lacking to ensure that Forward Head Syndrome is the etiological factor.

## INTRODUCCIÓN

Los trastornos temporomandibulares (TTM) son un grupo de signos y síntomas que se asocian con la disfunción del sistema masticatorio, especialmente en la articulación temporomandibular (ATM) ^1^^-^^3^. Los signos más comunes son el dolor y la limitación en el rango de movilidad articular, pero también la presencia de ruidos articulares, las alteraciones del complejo disco-cóndilo, la incompatibilidad estructural de las superficies óseas, y el dolor irradiado hacia el oído y los dientes ^3^^,^^4^.

Se han realizado múltiples estudios para esclarecer la asociación entre TTM y postura, los cuales han concluido que existe una relación entre una postura cervical patológica y el desarrollo de TTM ^5^^,^^6^. La postura es la posición del cuerpo en el espacio y su finalidad es mantener el cuerpo en equilibrio, tanto en reposo como en movimiento. Viene determinado por el sistema neuromuscular que envía información visual, auditiva y propioceptiva ^7^. Tres curvaturas fisiológicas son las que mantienen el equilibrio del ser humano: la lordosis cervical, la cifosis torácica y la lordosis lumbar; estas, con el crecimiento del individuo, determinarán su postura, que estará plenamente desarrollada alrededor de los 11 años y se establecerá a lo largo de la vida ^7^. Los malos hábitos, como la mala postura durante el uso de dispositivos tecnológicos, el uso excesivo de *smartphones*, el mal control abdominal y el patrón de respiración oral pueden conducir a una posición de la cabeza hacia adelante, lo que tiene implicaciones importantes en todo el individuo, especialmente a nivel del sistema estomatognático ^8^. 

Se ha informado que existe una relación entre la función de las vías respiratorias y la postura de la cabeza a través de mecanismos neuromusculares centrales. Debido a que la respiración es la función principal de soporte vital, los sistemas que rodean la vía aérea deben adaptarse para que puedan cumplir su función en condiciones desfavorables ^9^. Una obstrucción en las vías aéreas superiores dará lugar a un patrón de respiración oral; para ello, la mandíbula descenderá y la tensión del músculo suprahioideo disminuirá. Esto hace que el hueso hioides pierda su fijación y se posicione más hacia atrás y hacia abajo, lo cual reduce la entrada de aire a nivel faríngeo, por lo que el individuo deberá adelantar aún más su cabeza para satisfacer la demanda de oxígeno ^9^^,^^10^. Lo señalado generará una extensión dorsal de la cabeza en las vértebras cervicales de C1-C3, junto con la flexión de las vértebras cervicales inferiores de C4 a C7, lo que aumentará la lordosis cervical ^9^. Esto puede conducir a diversas alteraciones que conforman el síndrome de la cabeza adelantada (SCA). 

Entre las características clínicas del SCA se han descrito el acortamiento de los músculos suboccipital, cervical posterior, trapecio superior y esplenio; la hiperextensión de la columna cervical y el aumento de la presión sobre la articulación atlantoidea occipital, que genera un aumento de la patología compresiva en la zona; la posición de los hombros hacia adelante; el control deficiente de las escápulas, y los cambios en el nivel de los músculos de la cabeza y el cuello ^8^. El propósito de este estudio de revisión es detallar las amplias implicaciones que genera la adopción de una postura de cabeza adelantada y demostrar su relación con el desarrollo de TTM.

## MATERIALES Y MÉTODOS

Para este estudio se realizó una búsqueda bibliográfica en PubMed, Cochrane Library y EMBASE, delimitada de enero de 2010 a enero de 2022, para conocer la literatura relacionada con los efectos del síndrome de cabeza adelante en el desarrollo de trastornos temporomandibulares. 

Se utilizó una combinación de los siguientes términos MESH para la búsqueda: “posición de la cabeza adelantada”, “síndrome de la cabeza adelantada”, “postura”, “cervical”, “trastornos de la articulación temporomandibular”, “trastornos de la ATM”, “disfunción de la articulación temporomandibular”, “síndrome de la articulación temporomandibular”, “síndrome de la articulación temporomandibular” y “trastornos craneomandibulares”.

Se utilizaron revisiones sistemáticas de ensayos controlados aleatorios, ensayos controlados aleatorios individuales, revisiones sistemáticas de estudios de cohortes, estudios de cohortes individuales, revisiones sistemáticas de estudios de casos y controles, y estudios de casos y controles individuales. Sin embargo, se descartaron los estudios basados en opiniones de expertos y series de casos clínicos de baja calidad.

Esta revisión siguió el protocolo PRISMA para seleccionar todos aquellos ítems que se consideran relevantes, así como el sistema de análisis PICO System.

Dos revisores, de forma independiente, buscaron y seleccionaron artículos relevantes en las bases de datos mencionadas. No hubo desacuerdo en los estudios seleccionados.

## RESULTADOS Y DISCUSIÓN

### Asociación entre los trastornos temporomandibulares y el síndrome de cabeza adelantada

Se ha descrito en la literatura la asociación directa entre la postura y los TTM. Así, autores como Chaves *et al*. ^11^ encontraron una fuerte evidencia en los cambios posturales craneocervicales que generan TTM de origen miógeno. En la misma línea, Hong *et al*. ^12^ describieron que el grupo de TTM con SCA tenía un dolor cervical autoinformado más fuerte que el grupo de TTM miofascial sin SCA, y también con respecto al grupo de control.

En controversia, Cuenca y Martínez ^6^ describieron que los pacientes con TTM no presentaban alteraciones posturales en la región cervical en comparación con los pacientes asintomáticos. A su vez, Addu *et al*. ^13^ determinaron que la postura de la cabeza no tenía un papel significativo en la causalidad de TTM.

Por otro lado, López-de-Uralde *et al*. ^14^ y Khan *et al*. ^15^ midieron la actividad electromiográfica de varios músculos en dos tipos de grupos: con SCA y sin SCA (grupo control). Encontraron que, en el grupo con SCA, el trapecio superior y el esternocleidomastoideo (SCM) habían aumentado significativamente la actividad EMG con respecto al grupo de control.

Si se evalúa el grosor del SCM, Hong *et al*. ^12^ mencionan que fue significativamente mayor en el grupo con SCA que en el grupo control. Mientras tanto, los músculos *rectus capitis posterior* (RCP) y *obliquus capitis superior* (OCS) fueron mayores en los participantes con SCA en comparación con los controles, pero no son significativos.

Con respecto a los cambios degenerativos de la ATM, Kang *et al*. ^16^ afirman que el SCA se desarrolló más en pacientes con osteoartritis progresiva de la mandíbula que en los que presentaban osteoartritis y sin cambios degenerativos mandibulares.

### Tratamiento del síndrome de cabeza adelantada

Se han descrito diferentes posibles tratamientos del SCA. Uno de ellos fue descrito por Paço *et al*. ^17^^,^^18^ en pacientes que padecían clase II esquelética. Mediante tratamiento de ortodoncia, se corrigió la postura craneocervical en decúbito prono, para volver a valores basales. Otro estudio de tratamiento fue el de Thakur *et al*. ^19^, quienes compararon dos tipos de tratamiento: a un grupo de pacientes se les realizó entrenamiento mediante fuerza de los flexores profundos del cuello con esfigmomanómetro, mientras que al segundo grupo se le aplicó estiramiento de los músculos flexores profundos del cuello con la ayuda de una Theraband verde. Encontraron una mejora estadísticamente significativa de la distancia de deslizamiento escapular (SLD) en el grupo en el que se aplicaron estiramientos, mientras que en el grupo que se ejercitó con Theraband se demostró una mejoría estadísticamente significativa para la discinesia escapular, y mejoraron la fuerza del cuello y la propiocepción del hombro ^19^.

Oh *et al*. ^20^ realizaron el tratamiento del SCA mediante *neurofeedback*, sin mostrar diferencias significativas en el soporte de peso anterior y el ángulo de flexión cervical del grupo de entrenamiento con *neurofeedback* y el grupo de control.

### Patrón muscular de los pacientes con síndrome de cabeza adelantada

Se ha mencionado la diferencia de fuerza o activación de ciertos músculos en pacientes con SCA. Khan *et al*. ^14^ realizaron mediciones de EMG en el trapecio superior y SCM observaron un aumento de la actividad en pacientes con SCA en comparación con los controles. De este modo, coincide con Bokaee *et al*. ^18^, quienes describieron una mayor delgadez del SCM en pacientes con SCA y creyeron que esto podría deberse a la actividad insuficiente de los músculos flexores cervicales profundos. A pesar de esto, ha habido controversia en la literatura científica sobre el grosor de los músculos en pacientes con TTM. Hay varios estudios que mencionan que la diferencia de grosor de los músculos en el TTM no es significativa en comparación con los controles, pero estos estudios han evaluado en su mayoría TTM con un componente muscular leve a moderado ^21^. Aunque en el estudio de Ariji *et al*. ^22^ demostró que había diferencias significativas en el grosor y la fuerza de contracción entre los controles y los pacientes con TTM, y fue mayor en el grupo de DTM con dolor durante 4 días a la semana y dolor por presión de 3/3 en varios músculos del sistema masticatorio. Esto puede indicar que, en los TTM severos, el grosor de los músculos involucrados puede ser mayor si están activos o menor en el grupo inhibido ^23^.

### Características de la ATM y cuello de los pacientes con síndrome de cabeza adelantada

Con respecto a la ATM y sus componentes articulares, Kang *et al*. ^16^ observaron que, en los pacientes con osteoartrosis progresiva de la ATM, la SCA se volvió más notoria en comparación con el grupo con la osteoartrosis no progresiva. De este modo, se podría decir que hay mayores cambios degenerativos de la ATM en los pacientes con SCA. Sin embargo, hay que destacar que el estudio se realizó en una muestra de personas mayores, por lo que los cambios degenerativos se pueden deber a otras condiciones o al propio paso de la edad. 

Con relación a la discapacidad cervical y del cuello, Cuenca y Martínez describieron una asociación moderada entre la postura de la cabeza y los TTM ^6^, lo que puede deberse a que los estudios se llevaron a cabo en pacientes con TTM crónicos, lo que puede conducir a una sensibilización central, por lo que serían necesarios más estudios objetivos para analizar esta área.

Con respecto a la afectación de los músculos del cuello, Lee ^24^ demostró que el estiramiento isométrico del músculo cervical extensor es más beneficioso para la restauración del desequilibrio neuromuscular que la del flexor en personas con SCA.

Se ha descrito la existencia de una relación directa entre el dolor de cuello y los pacientes con SCA. Así, Sun *et al*. ^26^ y Chiou *et al*. ^26^ determinaron que, en pacientes más jóvenes con SCA y con lordosis cervical reducida, el dolor de cuello era de tipo espontáneo. Pero se necesitan más estudios para determinar el mecanismo del dolor de cuello en pacientes con postura de cabeza hacia adelante.

Generalmente, este dolor localizado en el cuello y la espalda se suele manifestar más a nivel del trapecio derecho, pero no tanto en el izquierdo ^27^. Kim demostró que, según el grado SCA, se detectaron cambios en los índices de discapacidad del cuello. Sin embargo, incluso un aumento en el ángulo de inclinación de la cabeza hacia adelante no condujo a una postura de hombros redondeados. Por lo tanto, mantener una postura adecuada puede prevenir el síndrome de dolor postural, la discapacidad funcional y la deformidad postural ^28^^,^^29^.

Yip *et al*. ^30^ demostraron la correlación negativa moderada entre el ángulo craneovertebral (CV) y la discapacidad del cuello. Los pacientes con ángulo CV pequeño tienen una mayor postura de la cabeza hacia adelante, y cuanto mayor es, aumenta la discapacidad.

## CONCLUSIONES

Hasta la fecha, falta evidencia científica respecto de la afectación según género y edad. Las limitaciones encontradas en la literatura se centran sobre todo en la aleatorización de las muestras y la unificación de criterios diagnósticos.

Existe una relación clínica relevante entre TMD y SCA. Esta asociación parece ser mayor en el componente muscular que en el articular, y se ven más afectados los músculos esternocleidomastoideos, trapecio y occipitales. Además, se incluye como un problema importante en estos pacientes el cuadro doloroso, que provoca disfunción craneocervical. Se han descrito diferentes tipos de tratamiento para el SCA, como la fisioterapia, que se puede acompañar con ortodoncia o cirugía, en los casos que lo necesiten. La fisioterapia resulta fundamental para restaurar el sistema completo, pero debe ir acompañada por un tratamiento multidisciplinar.

## Fuente de financiamiento:

Autofinanciado
